# Social and Behavioral Factors, Oral Health, and Dental Care Utilization

**DOI:** 10.1093/geroni/igaa057.2899

**Published:** 2020-12-16

**Authors:** Bei Wu, Stephen Shuman, Elisa Ghezzi

## Abstract

Oral health status and dental care utilization is strongly associated with social and behavioral factors and health outcomes. The five papers in this symposium address how several of these factors affect oral health and dental care among diverse groups of older adults. Using data from the Health and Retirement Study, the first paper examined the impact of early childhood disadvantages on oral health in later life among adults age 51 and above in the U.S. The second paper used large-scale epidemiological data that addressed the relationship between acculturation and subsequent oral health problems. It also tested the moderating role of neighborhood disorder in such a relationship among older Chinese Americans. The third paper demonstrated the importance of examining different pathways among foreign-born and native-born Chinese older adults with regard to offspring’s support on their oral health outcomes. While increasing evidence shows that cognitive function is associated with oral health, limited studies have been conducted to examine the impact of cognitive impairment, e.g., Alzheimer’s Disease (AD) and related dementias (RD), on dental care utilization and costs in older adults. The fourth paper aimed to address this knowledge gap. Results showed that AD and RD had different impacts on different types of dental care utilization and costs. The fifth paper further displayed that individuals with cognitive impairment face a significant challenge in handling dental-related medications. This symposium provides policy and clinical implications on improving oral health and dental care utilization among older adults in the U.S. Oral Health Interest Group Sponsored Symposium.

